# miR‐495 promotes intestinal epithelial cell apoptosis through downregulation of Sphingosine‐1‐phosphate

**DOI:** 10.14814/phy2.70021

**Published:** 2024-09-11

**Authors:** Ruiyun Li, Cassandra Cairns, Ting‐Xi Yu, Rao Jaladanki, Claire M. Dodson, Hee Kyoung Chung, Lan Xiao, Jian‐Ying Wang, Douglas J. Turner

**Affiliations:** ^1^ Baltimore Veterans Affairs Medical Center Baltimore Maryland USA; ^2^ Department of Surgery, Cell Biology Group University of Maryland School of Medicine Baltimore Maryland USA; ^3^ Ohio University Heritage College of Osteopathic Medicine Athens Ohio USA; ^4^ Department of Pathology, Cell Biology Group University of Maryland School of Medicine Baltimore Maryland USA

**Keywords:** IEC‐6 cells, intestinal mucosal injury, microRNAs, sphingosine kinase 1, Sphingosine‐1‐phosphate (S1P)

## Abstract

Many pathological conditions lead to defects in intestinal epithelial integrity and loss of barrier function; Sphingosine‐1‐phosphate (S1P) has been shown to augment intestinal barrier integrity, though the exact mechanisms are not completely understood. We have previously shown that overexpression of Sphingosine Kinase 1 (SphK1), the rate limiting enzyme for S1P synthesis, significantly increased S1P production and cell proliferation. Here we show that microRNA 495 (miR‐495) upregulation led to decreased levels of SphK1 resultant from a direct effect at the *SphK1* mRNA. Increasing expression of miR‐495 in intestinal epithelial cells resulted in decreased proliferation and increased susceptibility to apoptosis. Transgenic expression of miR‐495 inhibited mucosal growth, as well as decreased proliferation in the crypts. The intestinal villi also expressed decreased levels of barrier proteins and exaggerated damage upon exposure to cecal ligation‐puncture. These results implicate miR‐495 as a critical negative regulator of intestinal epithelial protection and proliferation through direct regulation of SphK1, the rate limiting enzyme critical for production of S1P.

## 1 INTRODUCTION

Maintenance of intestinal epithelial barrier integrity is vital for survival of mammalian organisms; preservation of mucosal integrity is vital for many intestinal functions and is orchestrated in the region with the highest turnover rate. Cells proliferate in the intestinal crypts and rapidly migrate to the tips of the villus all the while maintaining an intact barrier to prevent toxic substances in the intestinal lumen from translocating and ultimately entering the bloodstream. As the cells migrate, they also undergo differentiation and eventual apoptosis. This process is extremely complex, and the exact mechanisms underlying the preservation of intestinal epithelial integrity are incompletely understood.

Sphingosine‐1‐phosphate (S1P) is a ubiquitous bioactive lipid mediator that has been previously shown to serve as a critical regulator of inflammation, paracellular permeability, apoptosis, and cell proliferation (Greenspon et al., 2011; Sun et al., 2023). S1P is generated through the metabolism of cell membranes as well as through conversion from dietary sphingomyelin, and its levels are tightly regulated. It can exert downstream effects through actions as a ligand at cell surface receptors, but also as a direct intracellular signaling moiety. It serves a variety of biological functions secondary to its ability to function intracellularly and extracellularly on five different G‐protein coupled S1P receptors as a second messenger ligand (Chen et al., 2022; Jiang et al., 2013). S1P is tightly regulated, most particularly through its generation from sphingosine kinases, especially Sphingosine Kinase 1 (SphK1), and it has been shown that increased activity of SphK1 can lead to positive regulatory effects through increased concentration of S1P. We have shown previously that increased expression of SphK1 has profound stimulatory effects on S1P levels and promotes proliferation of the intestinal epithelial cells (IECs) (Jiang et al., 2013). However, the source of regulation of SphK1 itself has not yet been described.

Micro‐ribonucleic acids (miRNAs) are small noncoding RNAs that exhibit a variety of cellular effects through direct binding to specific mRNAs that can promote targeted degradation of that mRNA or inhibit its translation. miRNAs have been shown to play critical roles in multiple physiological and pathological processes, including with intestinal epithelial barrier function. We and others have reported on several miRNAs that contribute to apoptosis regulation, and mucosal epithelial restitution (Chung et al., 2015; Yang et al., 2019; Zhuang et al., 2013). We hypothesized that SphK1 itself was regulated by a miRNA as well.

miR‐495 is located on chromosome 14q32.31. It is cleaved into miR‐495‐3p and miR‐495‐5p from 3′‐ and 5′‐ strands of the pre‐miR‐495 stem‐loop structure. (Chen et al., 2017) We have previously identified differential expression of miR‐495 during polyamine depletion using miRNA array analysis, leading us to speculate its role in cell proliferation and healing (Xiao et al., 2011). Though miR‐495 has been shown to exhibit effects in various pathologic states (Fang et al., 2020; Zhang et al., 2018; Zhao et al., 2019), its role in the gastrointestinal tract—and the intestinal epithelium in particular—has been incompletely defined. Here, we investigated whether miR‐495 and SphK1 interact in IECs in vitro, followed with development of transgenic mice that specifically overexpress miR‐495 in the intestinal mucosa. In these models, we ultimately show that miR‐495 acts as a negative regulator of intestinal epithelial growth by inhibiting production of SphK1.

## 2 MATERIALS AND METHODS

### 2.1 Chemicals and cell culture

Dulbecco's modified Eagle's Medium (DMEM #11965092) and dialyzed fetal bovine serum (FBS #26400044) were obtained from Invitrogen (Carlsbad, CA), and biochemicals were from Sigma (St. Louis, MO). These experiments utilized differentiated IECs (IEC‐6) and stable Cdx‐2 transfected IEC‐6 cells (IEC‐Cdx2L1). The IEC‐6 cells lines are derived from normal rat intestinal crypt cells and were purchased from the American Type Culture Collection (CRL‐1592). IEC‐Cdx2L1 were gifted from Dr. P. G. Traber, University of Pennsylvania, Philadelphia, PA. These cells are nontumorigenic and retain characteristics of undifferentiated IECs (Greenspon et al., 2011). The IEC‐6 cells were maintained in DMEM supplemented with 5% heat‐inactivated FBS and antibiotics. Antibodies recognizing SPHK1 (INV‐PA514068), Caspase‐3 (CST‐9662S), PCNA (CST‐9661S), E‐cadherin (BD #610182), Claudin‐3, HSC70 (AB‐19136), and GAPDH (SC‐47724) were obtained from Cell signal Technology, Santa Cruz Biotechnology, BD Biosciences, and Invitrogen. Secondary antibodies conjugated to horseradish peroxidase were purchased from Sigma‐Aldrich.

### 2.2 Generation of miR495‐Tg mice

To generate miR‐495 transgenic (miR495‐Tg) mice, a 774–base pair (bp) fragment, including the mouse *miR‐495* locus on chromosome 12 (313‐bp primary miR‐495 sequence) and human β‐globinintron (216‐bp 5′ upstream sequence and 245‐bp 3′ sequence), was cloned into thepIRES‐AcGFP1‐Nuc vector by using an miR‐495 cloning primer set. A33 is a membrane antigen expressed in colonic and small bowel epithelium, and therefore the A33 promoter was used to drive intestinal epithelial tissue‐specific overexpression of the genomic miR‐495 precursor, as reported by others (Cafferata et al., 2009; Flentjar et al., 2007).

Transgenic founders on a pure C57BL/6J background were established by pronuclear injection at the University of Harvard Genome Modification Facility. Genotyping was performed by polymerase chain reaction (PCR) in deoxyribonucleic acid (DNA) extracted by tail clippings to identify the first generation of recombinant mice with the A33‐miR‐495 primer set. Two founders were obtained, and they were further characterized for the transmission or the expression of the transgene. Transgenic colonies were subsequently established. Male miR495‐Tg mice mated with wild‐type (WT) female mice to generate miR495‐Tg mice and their WT littermates for experiments. Representative results from two independent founders are reported here and compared with those obtained from littermate controls.

### 2.3 Animal experiments

All experiments were approved according to animal experimental ethical guidelines by the University of Maryland Baltimore and Baltimore Veteran's Affairs Medical Center Institutional Animal Care and Use. Mice were housed and handled in a specific pathogen‐free area and cared for by trained technicians and veterinarians. They were fed ENVIGO Rodent Sterilizable Diet. Both male and female mice were used for all experiments. For body weight comparisons, pairs of both male and female transgenic versus littermate mice were used.

To examine gut mucosal growth, bromodeoxyuridine (BrdU) was incorporated in intestinal mucosa by intraperitoneal injection of 2 mg BrdU (CST‐5292S) in phosphate‐buffered saline. A 4‐cm small intestinal segment taken 0.5 cm distal to the ligament of Trietz and a segment of middle colon were collected 1 h after injection.

Cecal ligation and puncture (CLP) was induced as described: The ligated cecum was punctured with a 25‐gauge needle and slightly compressed with an applicator until a small amount of stool appeared. In sham‐operated animals, the cecum was manipulated, but without ligation and puncture.

### 2.4 Histological analysis

Dissected intestinal tissues were fixed in formalin and paraffin. Sections were stained with hematoxylin and eosin (H&E) for general histology. Slides were graded in a blinded fashion.

### 2.5 Assays of gut permeability in mice

FITC‐conjugated dextran dissolved in water (Sigma; 4KD; 600 mg/kg #60842–46‐8) was administered to mice via gavage as described (Xiao et al., 2016). Blood was collected 4 h thereafter via cardiac puncture. The serum concentration of the FITC‐dextran was determined using a plate reader with an excitation wavelength at 490 nm and an emission wavelength of 530 nm. The concentration of FITC‐dextran in sera was determined by comparison to the FITC dextran standard curve.

### 2.6 Western blot analysis

Whole cell lysates were prepared using 2% SDS, sonicated, and centrifuged at 4°C for 15 min. The supernatants were boiled for 5 min and size‐fractionated by SDS‐PAGE. After proteins were transferred onto nitrocellulose filters, the blots were incubated with primary antibodies. After incubations with secondary antibodies (Anti‐rat CST 7077S, Anti‐mouse SC‐2005, Anti‐rabbit SC‐2357), immunocomplexes were developed using chemiluminescence (TF Pierce ECL #32209). All antibodies used in this study were validated for species specificity. Primary antibody dilutions used for Western blots were diluted 1:1000 or 1:2000 and secondary antibodies were diluted 1:2000. Relative protein levels were analyzed by using Bio‐Rad Chemidoc We also used “Quantity tool” to determine the band intensity volume; the values were normalized with internal loading control GAPDH or HSC70.

### 2.7 Reverse transcription (RT) followed by polymerase chain reaction (PCR) and real‐time quantitative (q)PCR analysis

Total RNA was isolated from cells after different treatments by using RNeasy mini kit (#217004 Qiagen, Valencia, CA) and used in reverse transcription (RT) and PCR amplification reactions as described previously (Ying et al., 2019; Zhang et al., 2021). The levels of glyceraldehyde‐3‐phosphate dehydrogenase (GAPDH) PCR product were assessed to monitor the evenness in RNA input in RT‐PCR samples. Real‐time quantitative (q)PCR analysis was performed using 7500‐fast real‐time PCR systems with specific primers (TF #4369016), probes, and software (Applied Biosystems, Foster City, CA).

### 2.8 Biotin labeling

Biotin labeled miR‐495 was transfected into the cells and 24 h later whole‐cell lysates were collected, mixed with Streptavidin‐Dynal beads (#60210 Invitrogen, Carlsbad, CA), and incubated at 4°C with rotation overnight. After the beads were washed thoroughly, the bead‐bound RNA was isolated and subjected for RT followed by Q‐PCR analysis. Input RNA was extracted and served as control.

### 2.9 Analysis of newly translated protein

New synthesis of SPHK1 protein was detected by Click‐iT protein analysis detection kit (Life technologies, Grand Island, NY) and performed following the company's manual with minor modification (Xiao et al., 2013). Briefly, cells were incubated in methionine free medium and then exposed to L‐azidohomoalanine (AHA). The cell lysate was mixed with the reaction buffer containing biotin/alkyne reagent and CuSO4 for 20 minutes. The biotin‐alkyne/azide‐modified protein complex was pulled down using paramagnetic streptavidin‐conjugated Dynabeads. The pull‐down material was resolved by 10% SDS‐PAGE and analyzed by Western immunoblotting analysis using the antibody against SPHK1 or GAPDH.

### 2.10 Intestinal organoid culture

Isolation and culture of primary enterocytes were conducted following methods described previously (Yu et al., 2020). Briefly, primary crypts were released from the small intestinal mucosa in mice, then the isolated crypts were mixed with Matrigel (#356234 Corning Inc, Corning, NY) and cultured in Mouse IntestiCult™ Organoid Growth Medium (#06000 STEMCELL Technologies Inc., Cambridge, MA). The growth of organoids was examined under phase contrast microscopy ZEISS LSM700 Fluorescence Confocal Microscope, (20× 40×).

### 2.11 Assessment of morphology and apoptosis

#### 2.11.1 Annexin V staining

After experimental treatments, cells were photographed with an inverted microscope before fixation (Nikon ECLIPSE 801(10x)). Annexin V staining for apoptosis was carried out by using a commercial apoptosis detection kit (Clontech ApoAlert Annexin V‐FITC Apoptosis Kit #630109) and performed according to the protocol recommended by the manufacturer. Briefly, cells were rinsed with 1 × binding buffer, and resuspended in 200 μL of 1 × binding buffer.

Annexin V (5 μL) was added on to the slide and incubated at room temperature for 10 min in the dark. Annexin‐stained cells were visualized and photographed under a fluorescence microscope using a dual filter, set for FITC, and the percentage of “apoptotic” cells was determined.

#### 2.11.2 Measurement of caspase‐3 activity

The caspase‐3 activity was measured by using the Caspase‐3 Colorimetric Assay kit (R&D Systems, Inc. Minneapolis, MN, U.S.A.) and performed according to the protocol recommended by the manufacturer. Briefly, cells were treated with TNF (tumor necrosis factor)‐α (R&D #410‐MT) and CHX (cycloheximide) (Sigma #C4359) for 3 h, washed with ice‐cold Dulbecco's‐PBS, and scraped from the dishes. The collected cells were washed with Dulbecco's‐PBS and then lysed in ice‐cold cell lysis buffer [50 mM Hepes (pH 7.4), 0.1% CHAPS, 1 mM DTT, 0.1 mM EDTA and 0.1% Nonidet P40]. The assay for caspase‐3 activity was carried out in a 96‐well plate. In each well, there was 50 μL of cell lysate (approx. 150 μg of total protein), 50 μL of reaction buffer [50 mM Hepes (pH 7.4), 0.1% CHAPS, 100 mM NaCl, 10 mM DTT and 1 mM EDTA], 5 μL of caspase‐3 colorimetric substrate, and a caspase‐specific peptide that is conjugated to a chromogen, *p*‐NA (*p*‐nitroanilide). The 96‐well plate was incubated at 37°C for 90 min, during which the caspase‐3 in the sample presumably cleaved the chromophore, p‐NA, from the substrate molecule. Absorbance readings at 405 nm were carried out after the incubation, with the caspase‐3 activity directly proportional to the color of the reaction. The protein level of each sample was determined by the method of Bradford (Kielkopf et al., 2020).

### 2.12 Statistics

Values are mean ± SD from three to six samples. Autoradiographic results were repeated three times. The significance of the difference between means was determined by analysis of variance. The level of significance was determined by using Duncan's multiple‐range test (Harter, 1960).

## 3 RESULTS

### 3.1 miR‐495 interacts with SphK1 mRNA and represses its translation in intestinal epithelial cells

**FIGURE 1:**
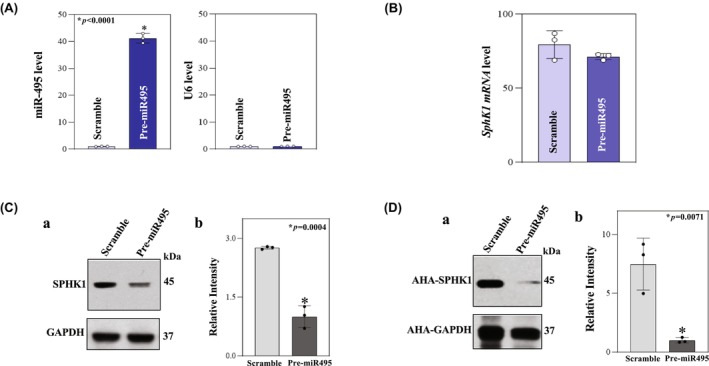
(A) Levels of miR‐495 and U6 endogenous control in cells transfected with pre‐miR495 for 48 h as measured by Q‐PCR analysis, *n* = 3. *p < 0.05 compared with cells transfected with control scrambled oligomer. (B) Levels of *SphK1* mRNA as examined by Q‐PCR analyses. (C) Decreased SPHK1 protein in miR‐495 overexpressed cells compared to scrambled control. Equal loading assessed with GAPDH levels (a), Quantitative analysis of the immunoblots with densitometry, *n* = 3 (b). (D) Levels of newly synthesized SPHK1 protein after ectopic miR‐495 overexpression via streptavidin‐conjugated Dynabeads (a). Quantitative analysis of the immunoblots with densitometry, *n* = 3 (b). All values represent mean ± SD.

Intestinal cell lines overexpressing miR‐495 were established in IEC‐6 cells. Real time quantitative PCR (Q‐PCR) analysis was used to verify a substantial increase in the levels of miR‐495 and no change in U6 control level after 48 h transfection (Figure 1A). Cells transfected with miR‐495 exhibited no significant change in *SphK1* mRNA with quantitative PCR but significant decrease in SPHK1 protein expression with Western Blot (Figure 1B,C). AHA incorporation measured by streptavidin bead pulldown assay demonstrated that low intensity of SPHK1 expression was due to decrease in newly synthesized protein (Figure 1D).

**FIGURE 2:**
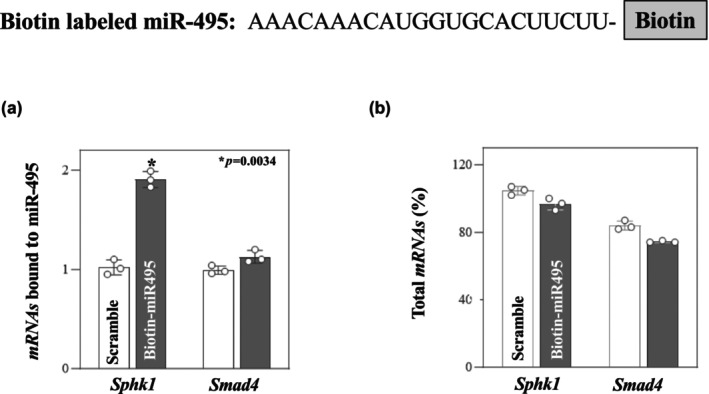
Biotinylated miR‐495 was transfected in Cdx‐2 cells for 24 h. *Sphk1* and *Smad4* levels with Biotin labeled miR‐495 (a) and total input mRNAs (b). *p‐value <0.05 compared with cells transfected with scramble, 2‐way ANOVA (n = 3). Values represent mean ± SD.

To assess the association between miR‐495 and the *SphK1* mRNA, RNA pull‐down assay using biotin labeled miR‐495 was used. The levels of *SphK1* mRNA were significantly enriched in the pulldown materials from cells transfected with biotin labeled miR‐495 but not cells transfected with a scrambled oligomer. This interaction of miR‐495 with *SphK1* mRNA is seemingly specific, as biotin labeled miR‐495 did not pull down the *SMAD4* mRNA (Figure 2a) This increase in *Sphk1* levels in the miR‐495 transfected cells was not a result of increased mRNA load, as depicted in Figure 2b.

### 3.2 Ectopic expression of miR‐495 induces apoptosis in IECs

**FIGURE 3:**
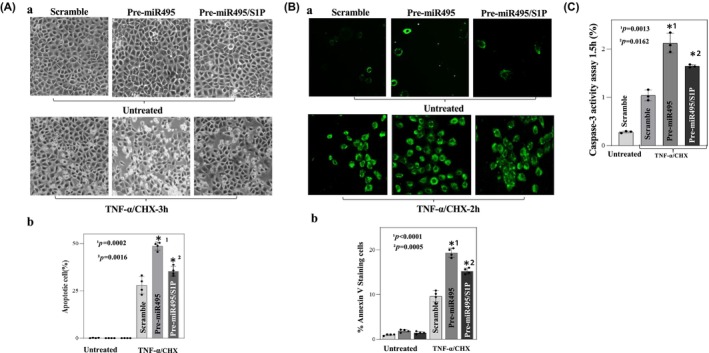
TNF‐α (20 ng/mL) plus CHX (25 μg/mL)—induced apoptosis after O/E miR‐495 and S1P treatment. (A) Representative images^+^ demonstrating apoptosis after treatment (a). Percentage of apoptotic cells after treatment, *n* = 4 (b). (B) Representative images^+^ of Annexin V staining for caspase‐3 depicting cell apoptosis (a), Percentage of apoptotic cells after treatment, *n* = 4 (b). (C) Caspase‐3 activity assay (*n* = 3). (Greenspon et al., 2011)Scramble vs. pre‐miR495, ^2^pre‐miR495 vs pre‐miR495/s1p. All values are mean +/− SD. ^+^Captured at 100× magnification.

To investigate the physiologic consequences of miR‐495 overexpression, we measured apoptosis in cells transfected with miR‐495. Microscopically, cells treated with miR‐495 exhibited increased susceptibility to apoptosis that improved with S1P supplementation (Figure 3A). miR‐495 overexpressing cells also demonstrated an increase in Caspase‐3 protein activity (Figure 3B). This effect was partially improved with synchronous administration of exogenous S1P. We next quantified susceptibility to apoptosis under these same conditions with Annexin staining for Caspase‐3. Following 3 h of treatment with TNF‐α/CHX, cells overexpressing miR‐495 showed significant increased susceptibility to apoptosis, and this was partially reversed with concomitant exposure to S1P (Figure 3C).

### 3.3 Validation of miR‐495 transgenic mice (Figure 4)

**FIGURE 4:**
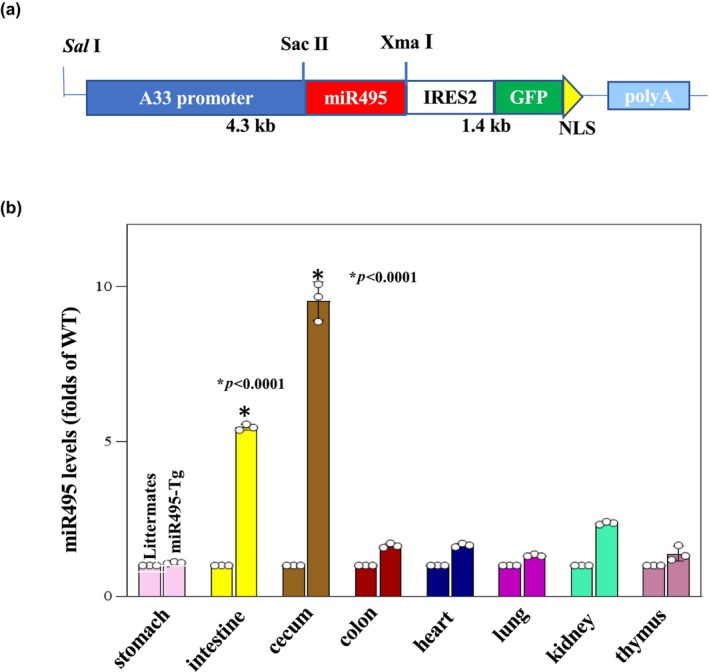
(a) A33‐miR495 IRES2‐GFP‐NLS. (b) Levels of miR‐495 in various mouse tissues as measured by Q‐PCR analysis (*n* = 3 animals). Values are mean +/− SD. *p < 0.05 compared with littermates.

Systemic miR‐495 overexpression was established in transgenic mice as previously described. Levels of miR‐495 were measured in various tissues with quantitative PCR. Compared to littermates, statistically significant elevations of miR‐495 were identified in the intestine and cecum of transgenic mice (Figure 4).

### 3.4 miR‐495 overexpression inhibits growth of small intestinal mucosa in transgenic mice

**FIGURE 5:**
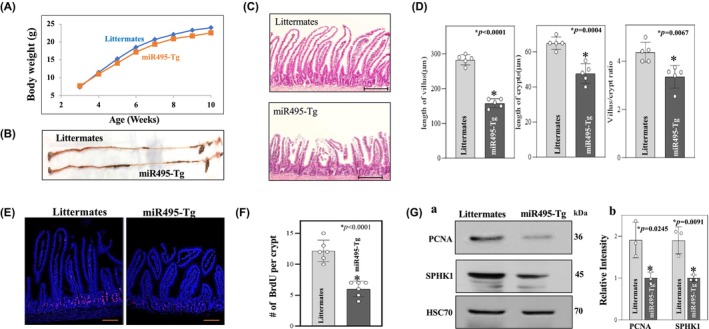
MiR‐495 inhibits growth of small intestinal mucosa. (A) Body weight of littermates and miR495‐Tg mice (*n* = 10 males). (B) Representative gastrointestinal gross morphology. (C) Representative photomicrographs of hematoxylin and eosin (H/E). Scale bar: 100 μm. (D) Changes in the length of villi (left), crypt (middle) and villus: Crypt ratio (*n* = 5 males). (E) Representative images of proliferating cells in small intestinal crypts as measured by BrdU labeling (red). Scale bars: 50 μm. (F) Summarized BrdU positive ceIls per crypt (*n* = 6 males). (G) Immunoblots of PCNA, SPHK1 and HSC70 in the intestinal mucosa (a). Quantitative analysis of the immunoblots as densitometry, *n* = 3 (b). *p < 0.5 from unpaired *t*‐test. All values are mean +/− SD.

To determine the effect of miR‐495 overexpression in vivo, we examined samples of small intestine from miR495‐Tg mice. Despite no significant change in body weight or gastrointestinal gross morphology (Figure 5A,B), miR495‐Tg mice exhibited shortened villi length, crypt length, and villus: crypt ratio on histologic examination (Figure 5C,D). miR‐495 transgenic mice also showed less BrdU staining in the crypts signifying decreased epithelial proliferation (Figure 5E,F). SPHK1 and PCNA protein expression were both decreased in the intestinal mucosa of miR‐495 transgenic mice, indicating both a decrease in SPHK1 expression as well as decreased cellular proliferation overall (Figure 5G).

### 3.5 Organoids derived from miR495‐Tg mice demonstrate impaired barrier function

**FIGURE 6:**
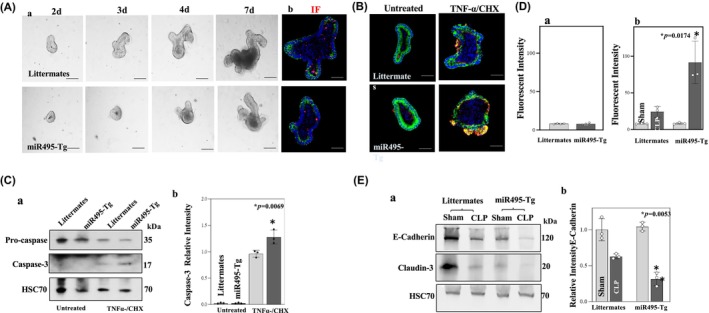
(A) Organoid growth in (a) Bright field microscopy; Proliferating cells in intestinal organoids as measured by BrdU labeling (Red; Scale bar: 50 μm) (b). (B) Immunostaining of Caspase‐3 (red, Scale bar: 50 μm) in organoids treated with TNF‐α/CHX. (C) Western blot of protein expression in organoids as compared to HSC70 loading control (a) with quantitative analysis of the immunoblots with densitometry, *n* = 3. *p < 0.05, miR495‐Tg versus littermates after TNF‐a/CHX treatment (b). (D) Basal level in FITC‐dextran paracellular permeability in intestinal mucosa, littermates versus miR495‐Tg (a). Epithelial barrier function as indicated by change in FITC‐dextran paracellular permeability in intestinal mucosa, (*n* = 4). *p < 0.05 of miR495‐Tg versus littermates after CLP (b). (E) Immunoblot of E‐cadherin and Claudin‐3 level in the intestinal mucosa of mice exposed to CLP for 24 h (a). Quantitative analysis of the immunoblots as densitometry, (*n* = 3) *p < 0.05 (b). All values mean ± SD.

To further categorize the influence of miR‐495 overexpression on the intestinal epithelium, organoids were grown from the small bowel of miR495‐Tg mice. miR495‐Tg organoids demonstrated repressed growth rate compared to littermates as quantified by growth area (Figure 6A). miR‐495 organoids also demonstrated an increased staining for Caspase‐3 after treatment with TNF‐α/CHX (Figure 6B) suggesting increased susceptibility to apoptosis.

### 3.6 miR495 mice show increased permeability to pathological stimuli

Cecal ligation and puncture (CLP) was performed in mice to assess intestinal response to sepsis conditions. miR495‐Tg mice demonstrated increased paracellular susceptibility following CLP compared to littermates as measured by FITC‐dextran permeability (Figure 6D). Western Blot analysis confirmed that miR495‐Tg mice demonstrated decreased expression of barrier proteins including E‐cadherin and Claudin‐3 after CLP (Figure 6E), and increased expression of Caspace‐3 (Figure 6C) when compared to littermates (Figure 6C).

## 4 DISCUSSION

S1P is known to have a proliferative effect in IECs (Jiang et al., 2013), but its regulation is poorly understood. In this novel study, we present evidence that miR‐495 overexpression is associated with decreased levels of SPHK1 protein as a result of a direct effect at the *SphK1* mRNA, ultimately culminating in decreased cell proliferation. Furthermore, miR‐495 overexpression increases susceptibility of cells to apoptosis during cellular stress and increases permeability of the intestinal epithelium via lowering levels of barrier proteins. Importantly, this effect can be partially reversed with exposure to S1P. In sum, this work suggests that miR‐495 exhibits negative effects on the intestinal epithelial barrier by inhibiting S*phK1* mRNA translation and ultimately decreasing SPHK1 production.

miR‐495 has been demonstrated to have differing effects in various organ systems (Wang et al., 2018; Ying et al., 2019; Zhang et al., 2021), and its expression is altered in many human cancers, where it can act as a tumor suppressor. It has been implicated in chemotherapeutic resistance and is under investigation as a prognostic marker (He et al., 2019; Huldani et al., 2023). In the intestine, the roles of miR‐495 are variable and at times contradictory. In colorectal cancer, miR‐495 appears to inhibit cancer cell proliferation and migration by targeting HMGB1 (Zhang et al., 2022). In inflammatory bowel disease, S1P has proven to be a valuable target whereby blocking the S1P pathway exhibits a therapeutic immunomodulatory effect (Liu et al., 2023). Conversely, S1P plays a protective role in sepsis and its levels are systemically decreased during sepsis when compared to healthy controls (Liu et al., 2023).

Interestingly, miR‐495 was recently shown to be differentially expressed in mouse models of sepsis. This study helps elucidate another possible mechanism by which miRNAs regulate the body's response to sepsis, and provides a potential therapeutic pathway to combat the increase in gut permeability commonly seen in septic states that leads to devastating bacterial translocation from the gut and systemic disease (Caidengbate et al., 2023). Taken together, these findings demonstrate the complex and sometimes contradictory regulatory networks involving miR‐495 in the intestinal epithelium. It is apparent that further investigation into the full function of miR‐495 is necessary before it can be explored as a therapeutic target. In future studies we intend to measure S1P levels in the tissues of miR‐495Tg mice to further explore the phenotype of these mice.

A notable result of this work is that S1P has been shown to be protective against the anti‐proliferative and barrier dysfunction effects seen with miR‐495 overexpression. This is consistent with prior studies that have demonstrated S1P promoting proliferation and barrier function of IECs, possibly through increasing the translation of c‐Myc (Greenspon et al., 2011; Jiang et al., 2013).

## 5 CONCLUSION

miR‐495 exhibits negative effects on intestinal epithelial barrier growth and integrity by inhibiting *Sphk1* mRNA translation and ultimately decreasing S1P production.

## AUTHOR CONTRIBUTIONS

R.L., J.N.R., J.‐Y.W., and D.J.T. conceived and designed research; R.L., T.X.Y., H.K.C., C.M.D., performed experiments; R.L., C.A.C., L.X., analyzed data; R.L., C.A.C., T.X.Y., C.M.D., H.K.C., L.X., interpreted results of experiments; R.L. C.A.C., prepared figures; R.L., C.A.C., D.J.T., drafted manuscript; R.L., C.A.C., L.X., J.‐Y. W., D.J.T., edited and revised manuscript; R.L., J.‐Y.W., and D.J.T. approved final version of manuscript.

## FUNDING INFORMATION

This work was supported by Merit Review Awards from the US Department of Veterans Affairs (to D.J. Turner, J.N. Rao, and J.Y. Wang) and from the National Institutes of Health under Grants DK‐57819, DK‐61972, and DK‐68491 (to J.Y. Wang). C. Cairns was supported by the National Institutes of Health NIDDK T32 under Grant DK‐067872‐19 (awarded to Jean‐Pierre Raufman).

## CONFLICT OF INTEREST STATEMENT

None.

## ETHICS STATEMENT

This study was approved by the Institutional Biosafety Committee (IBC‐00000344), Institutional Review Board (HCR‐HP‐00040847‐14) and the Institutional Animal Care and Use Committee (IACUC) (#0222010) at the University of Maryland School of Medicine.

## Data Availability

Source data is not publicly available.

## References

[phy270021-bib-0001] Cafferata, E. G. , Macció, D. R. , Lopez, M. V. , Viale, D. L. , Carbone, C. , Mazzolini, G. , & Podhajcer, O. L. (2009). A novel A33 promoter‐based conditionally replicative adenovirus suppresses tumor growth and eradicates hepatic metastases in human colon cancer models. Clinical Cancer Research, 15(9), 3037–3049. 10.1158/1078-0432.CCR-08-1161 19336523

[phy270021-bib-0002] Caidengbate, S. , Akama, Y. , Banerjee, A. , Mokmued, K. , Kawamoto, E. , Gaowa, A. , McCullough, L. D. , Shimaoka, M. , Lee, J. , & Park, E. J. (2023). MicroRNA profiles in intestinal epithelial cells in a mouse model of sepsis. Cells, 12(5), 726. 10.3390/cells12050726 36899862 PMC10001189

[phy270021-bib-0003] Chen, H. , Wang, J. , Zhang, C. , Ding, P. , Tian, S. , Chen, J. , Ji, G. , & Wu, T. (2022). Sphingosine 1‐phosphate receptor, a new therapeutic direction in different diseases. Biomedicine & Pharmacotherapy, 153, 113341. 10.1016/j.biopha.2022.113341 35785704

[phy270021-bib-0004] Chen, H. , Wang, X. , Bai, J. , & He, A. (2017). Expression, regulation and function of miR‐495 in healthy and tumor tissues. Oncology Letters, 13(4), 2021–2026. 10.3892/ol.2017.5727 28454357 PMC5403365

[phy270021-bib-0005] Chung, H. K. , Chen, Y. , Rao, J. N. , Liu, L. , Xiao, L. , Turner, D. J. , Yang, P. , Gorospe, M. , & Wang, J. Y. (2015). Transgenic expression of miR‐222 disrupts intestinal epithelial regeneration by targeting multiple genes including Frizzled‐7. Molecular Medicine, 21(1), 676–687. 10.2119/molmed.2015.00147 26252186 PMC4749484

[phy270021-bib-0006] Fang, L. , Xu, X. F. , Lu, Y. , Wu, Y. Y. , & Li, J. J. (2020). MicroRNA‐495 attenuates proliferation and inflammatory response in rheumatoid arthritis fibroblast‐like synoviocytes through attenuating β‐catenin pathway. Journal of Biological Regulators and Homeostatic Agents, 34(3), 837–844. 10.23812/20-47-A-22 32677423

[phy270021-bib-0007] Flentjar, N. , Chu, P. Y. , Ng, A. Y. N. , et al. (2007). TGF‐betaRII rescues development of small intestinal epithelial cells in Elf3‐deficient mice. Gastroenterology, 132(4), 1410–1419. 10.1053/j.gastro.2007.02.054 17408644

[phy270021-bib-0008] Greenspon, J. , Li, R. , Xiao, L. , Rao, J. N. , Sun, R. , Strauch, E. D. , Shea‐Donohue, T. , Wang, J. Y. , & Turner, D. J. (2011). Sphingosine‐1‐phosphate regulates the expression of adherens junction protein E‐cadherin and enhances intestinal epithelial cell barrier function. Digestive Diseases and Sciences, 56(5), 1342–1353. 10.1007/s10620-010-1421-0 20936358 PMC4140085

[phy270021-bib-0009] Harter, H. L. (1960). Critical values for Duncan's new multiple range test. Biometrics, 16(4), 671. 10.2307/2527770

[phy270021-bib-0010] He, J. , Gong, C. , Qin, J. , Li, M. , & Huang, S. (2019). Cancer cell membrane decorated silica nanoparticle loaded with miR495 and doxorubicin to overcome drug resistance for effective lung cancer therapy. Nanoscale Research Letters, 14(1), 339. 10.1186/s11671-019-3143-3 31705398 PMC6841775

[phy270021-bib-0011] Huldani, H. , Alshahrani, S. H. , Almajidi, Y. Q. , Romero‐Parra, R. M. , Hjazi, A. , Alsaab, H. O. , Oudaha, K. H. , Hussien, B. M. , Ahmed, M. , & Fard, S. R. H. (2023). miR‐495‐3p as a promising tumor suppressor in human cancers. Pathology, Research and Practice, 248, 154610. 10.1016/j.prp.2023.154610 37307621

[phy270021-bib-0012] Jiang, P. , Smith, A. D. , Li, R. , Rao, J. N. , Liu, L. , Donahue, J. M. , Wang, J. Y. , & Turner, D. J. (2013). Sphingosine kinase 1 overexpression stimulates intestinal epithelial cell proliferation through increased c‐Myc translation. American Journal of Physiology. Cell Physiology, 304(12), C1187–C1197. 10.1152/ajpcell.00271.2012 23576579 PMC4073999

[phy270021-bib-0013] Kielkopf, C. L. , Bauer, W. , & Urbatsch, I. L. (2020). Bradford Assay for Determining Protein Concentration. Cold Spring Harbor Protocols, 2020(4), 102269. 10.1101/pdb.prot102269 32238597

[phy270021-bib-0014] Liu, J. , Di, B. , & Xu, L. L. (2023). Recent advances in the treatment of IBD: Targets, mechanisms and related therapies. Cytokine & Growth Factor Reviews, 71–72, 1–12. 10.1016/j.cytogfr.2023.07.001 37455149

[phy270021-bib-0015] Sun, G. , Wang, B. , Zhu, H. , Ye, J. , & Liu, X. (2023). Role of sphingosine 1‐phosphate (S1P) in sepsis‐associated intestinal injury. Frontiers in Medicine, 10, 1265398. 10.3389/fmed.2023.1265398 37746079 PMC10514503

[phy270021-bib-0016] Wang, X. , Jin, H. , Jiang, S. , & Xu, Y. (2018). MicroRNA‐495 inhibits the high glucose‐induced inflammation, differentiation and extracellular matrix accumulation of cardiac fibroblasts through downregulation of NOD1. Cellular & Molecular Biology Letters, 23(1), 23. 10.1186/s11658-018-0089-x 29760746 PMC5941488

[phy270021-bib-0017] Xiao, L. , Cui, Y. H. , Rao, J. N. , Zou, T. , Liu, L. , Smith, A. , Turner, D. J. , Gorospe, M. , & Wang, J. Y. (2011). Regulation of cyclin‐dependent kinase 4 translation through CUG‐binding protein 1 and microRNA‐222 by polyamines. Molecular Biology of the Cell, 22(17), 3055–3069. 10.1091/mbc.e11-01-0069 21737690 PMC3164454

[phy270021-bib-0018] Xiao, L. , Rao, J. N. , Cao, S. , Liu, L. , Chung, H. K. , Zhang, Y. , Zhang, J. , Liu, Y. , Gorospe, M. , & Wang, J. Y. (2016). Long noncoding RNA SPRY4‐IT1 regulates intestinal epithelial barrier function by modulating the expression levels of tight junction proteins. Molecular Biology of the Cell, 27, 617–626. 10.1091/mbc.E15-10-0703 26680741 PMC4750922

[phy270021-bib-0019] Xiao, L. , Rao, J. N. , Zou, T. , Liu, L. , Cao, S. , Martindale, J. L. , Su, W. , Chung, H. K. , Gorospe, M. , & Wang, J. Y. (2013). miR‐29b represses intestinal mucosal growth by inhibiting translation of cyclin‐dependent kinase 2. Molecular Biology of the Cell, 24(19), 3038–3046. 10.1091/mbc.e13-05-0287 23904268 PMC3784378

[phy270021-bib-0020] Yang, F. , Li, X. F. , Cheng, L. N. , & Li, X. L. (2019). Long non‐coding RNA CRNDE promotes cell apoptosis by suppressing miR‐495 in inflammatory bowel disease. Experimental Cell Research, 382(2), 111484. 10.1016/j.yexcr.2019.06.029 31251902

[phy270021-bib-0021] Ying, Y. , Mao, Y. , & Yao, M. (2019). NLRP3 Inflammasome activation by MicroRNA‐495 promoter methylation may contribute to the progression of acute lung injury. Molecular Therapy–Nucleic Acids, 18, 801–814. 10.1016/j.omtn.2019.08.028 31734560 PMC6861628

[phy270021-bib-0022] Yu, T. X. , Chung, H. K. , Xiao, L. , Piao, J. J. , Lan, S. , Jaladanki, S. K. , Turner, D. J. , Raufman, J. P. , Gorospe, M. , & Wang, J. Y. (2020). Long noncoding RNA H19 impairs the intestinal barrier by suppressing autophagy and lowering Paneth and goblet cell function. Cellular and Molecular Gastroenterology and Hepatology, 9(4), 611–625. 10.1016/j.jcmgh.2019.12.002 31862317 PMC7078540

[phy270021-bib-0023] Zhang, J. L. , Zheng, H. F. , Li, K. , & Zhu, Y. P. (2022). miR‐495‐3p depresses cell proliferation and migration by downregulating HMGB1 in colorectal cancer. World Journal of Surgical Oncology, 20(1), 101. 10.1186/s12957-022-02500-w 35354479 PMC8966301

[phy270021-bib-0024] Zhang, W. Q. , Feng, L. , Wang, H. J. , du, X. F. , Hao, X. L. , Li, Y. Z. , & Cheng, L. (2021). Inhibition of microRNA‐495 inhibits hypoxia‐induced apoptosis in H9c2 cells via targeting NFIB. European Review for Medical and Pharmacological Sciences, 25(1), 335–343. 10.26355/eurrev_202101_24399 33506922

[phy270021-bib-0025] Zhang, X. , Yang, Y. , & Feng, Z. (2018). Suppression of microRNA‐495 alleviates high‐glucose‐induced retinal ganglion cell apoptosis by regulating notch/PTEN/Akt signaling. Biomedicine & Pharmacotherapy, 106, 923–929. 10.1016/j.biopha.2018.07.018 30119264

[phy270021-bib-0026] Zhao, X. , Wang, T. , Cai, B. , Wang, X. , Feng, W. , Han, Y. , Li, D. , Li, S. , & Liu, J. (2019). MicroRNA‐495 enhances chondrocyte apoptosis, senescence and promotes the progression of osteoarthritis by targeting AKT1. American Journal of Translational Research, 11(4), 2232–2244. 31105831 PMC6511756

[phy270021-bib-0027] Zhuang, R. , Rao, J. N. , Zou, T. , Liu, L. , Xiao, L. , Cao, S. , Hansraj, N. Z. , Gorospe, M. , & Wang, J. Y. (2013). miR‐195 competes with HuR to modulate stim1 mRNA stability and regulate cell migration. Nucleic Acids Research, 41(16), 7905–7919. 10.1093/nar/gkt565 23804758 PMC3763549

